# Size isn't everything

**DOI:** 10.7554/eLife.07604

**Published:** 2015-04-17

**Authors:** Ronald E Ellis, Qing Wei

**Affiliations:** Department of Molecular Biology, Rowan University School of Osteopathic Medicine, Stratford, United Statesellisre@rowan.edu; Rowan University Graduate School of Biomedical Sciences, Stratford, United States

**Keywords:** sperm competition, sexual selection, cell migration, reproductive success, nematode, *C. elegans*

## Abstract

Male nematode worms may make larger sperm than hermaphrodite worms, but this is not the only reason that sperm from males have a competitive edge.

**Related research article** Hansen JM, Chavez DR, Stanfield GM. 2015. COMP-1 promotes competitive advantage of nematode sperm. *eLife* **4**:e05423. doi: 10.7554/eLife.05423

**Image** Nematode sperm (green) compete to enter structures called spermathecae

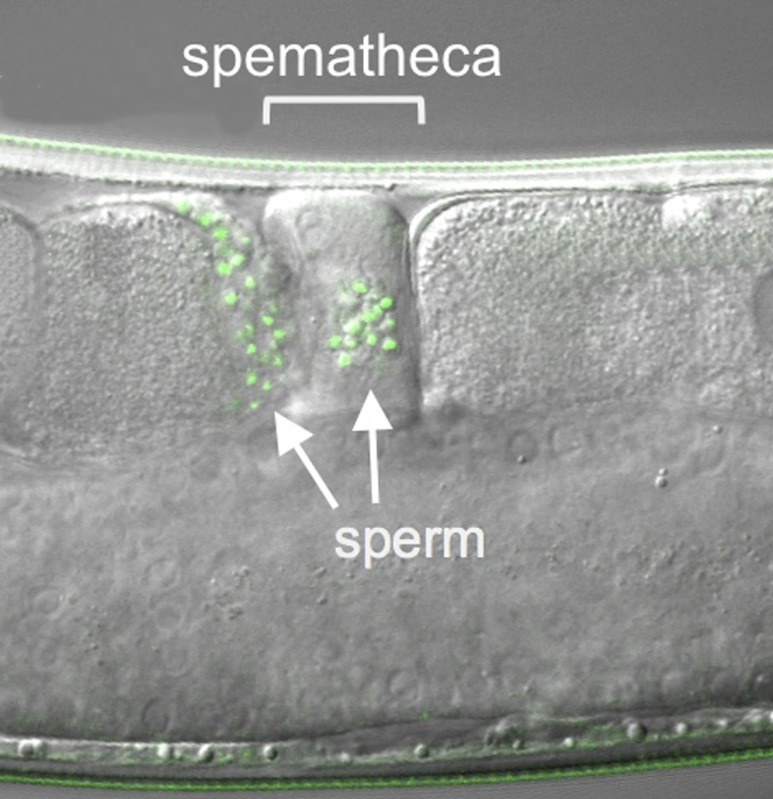

In his second major book, *The Descent of Man and Selection in Relation to Sex*, Charles Darwin showed that when animals compete for mates, sexual selection can drive many types of evolutionary change (Darwin, 1871). However, he did not notice that this competition continued even after animals had finished mating.

Sperm competition was only described a century later when groundbreaking work showed that if two (or more) males mate with a single female, their sperm compete for the chance to fertilize her egg cells or ‘oocytes’ (Parker, 1970). Since this process is carried out inside her reproductive tract, the female can influence the outcome, and the males can too, via chemicals released in their ejaculates. This competition can be lengthy and intense, particularly in animal species where females store sperm for later use.

The sperm of nematode worms resemble those of other animals in many ways, but they move by crawling rather than by swimming. The nematode *Caenorhabditis elegans* is ideal for studying sperm competition because its short lifespan and large brood size make the animals easy to work with in the laboratory. Furthermore, this worm's genetics have been well studied. Now, in *eLife*, Jody Hansen, Daniela Chavez and Gillian Stanfield—who are all at the University of Utah—have used the power of nematode genetics to transform our understanding of sperm competition (Hansen et al., 2015).

*C. elegans* has two sexes, males and hermaphrodites, both of which make sperm. Thus, several types of competitive interactions can be studied using these animals—the most important of which involves sperm from a male outcompeting sperm from a hermaphrodite to fertilize the hermaphrodite's own oocytes. A series of beautiful studies showed that the competitive advantage of male sperm can be explained, in part, because they are larger than hermaphrodite sperm (LaMunyon and Ward, 1995, 1998). Furthermore, comparisons between related species supported the idea that competition favored larger sperm in nematodes, and this model was confirmed by laboratory experiments (LaMunyon and Ward, 1999, 2002).

As a result, a simple model for how nematode sperm compete was proposed (Figure 1A; Ellis and Scharer, 2014). Inside a hermaphrodite, sperm are stored in the two structures called spermathecae and fight for positions near the maturing oocytes, where they will have the best chance of fertilization. Since each newly fertilized oocyte passes through the spermatheca, it dislodges many of the sperm, setting up a new round of competition to move into a good spot. Larger sperm appear to handle this intense competition better.

**Figure 1.**
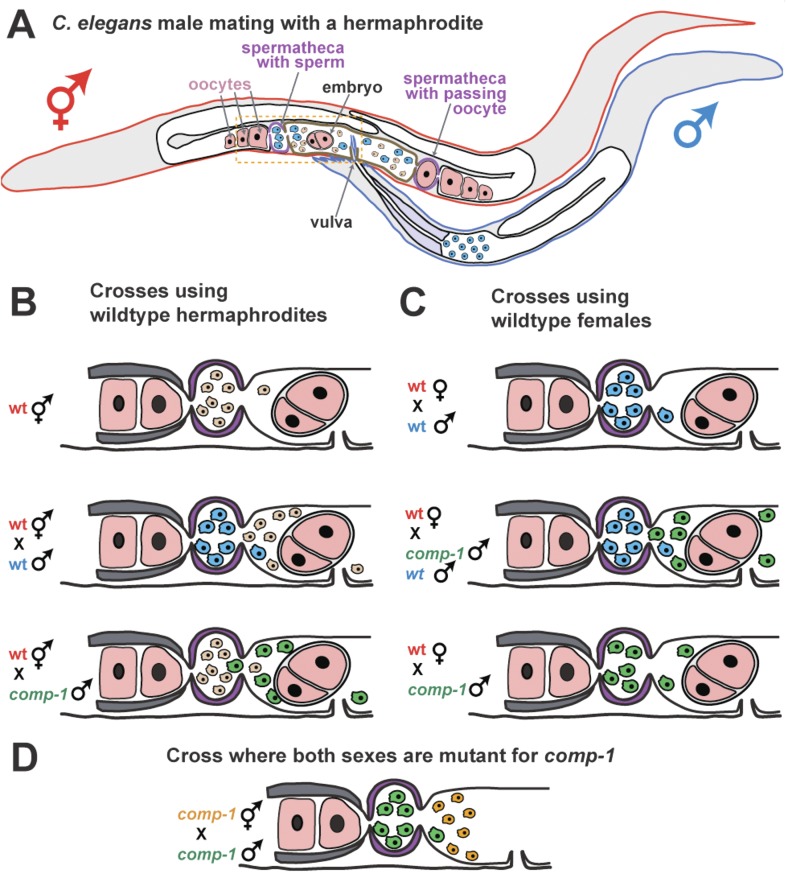
COMP-1 controls sperm competition in nematodes. (**A**) When a normal male (outlined in blue) mates with a hermaphrodite (red), his sperm (blue) are larger than the hermaphrodite sperm (pink) and are better able to compete for positions in the two spermathecae (purple). Sperm located in the spermathecae are in a better position to fertilize the hermaphrodite's oocytes. Some sperm and a young embryo are shown in the uterus (brown), and the passage of an oocyte through the spermatheca on the right has dislodged the sperm. The region inside the dashed box is expanded in **B**–**D**. (**B**) Male sperm can normally outcompete hermaphrodite sperm and enter the spermathecae (middle), but Hansen et al. show that *comp-1* mutant male sperm (green) are outcompeted by hermaphrodite sperm (bottom). (**C**) Furthermore, although *comp-1* sperm can fertilize female oocytes in the absence of competition (bottom), they cannot compete with normal male sperm (middle). (**D**) When both the male sperm (green) and hermaphrodite sperm (orange) are made by *comp-1* mutants, the larger male sperm have a competitive advantage. This shows that the COMP-1 protein and sperm size independently affect competition.

Hansen, Chavez and Stanfield took advantage of the fact that male sperm normally outcompete the hermaphrodite's own sperm, and looked for rare males whose sperm could not compete with hermaphrodite sperm. This approach identified the gene, *comp-1,* which they named for its role in sperm competition.

Sperm from males with a mutation in *comp-1* lose to those from normal hermaphrodites, even though they are larger (Figure 1B). And, the sperm from *comp-1* mutant males also lose to sperm from normal males following a double mating (Figure 1C). Nevertheless, *comp-1* mutant hermaphrodites can still self-fertilize, and *comp-1* mutant males can fertilize ‘true females’ (hermaphrodites that are unable to make their own sperm). This indicates that the mutant sperm work fine in the absence of competition. Thus, *comp-1* controls an aspect of sperm competition that is independent of size. This idea is supported by the fact that larger *comp-1* mutant sperm (produced by *comp-1* males) are still favored over smaller *comp-1* mutant sperm (produced by *comp-1* hermaphrodites) (Figure 1D).

Hansen, Chavez and Stanfield observed living worms and showed that *comp-1* mutant sperm did not migrate to the two spermathecae as quickly as expected, and certain in vitro tests revealed that *comp-1* sperm have shorter pseudopods (‘foot’-like projections that help cells to crawl). Since nematode sperm must crawl quickly towards the oocytes to compete, these defects suggest that the protein encoded by the *comp-1* gene is involved in sperm movement or guidance.

In animals, oocytes often provide chemical cues to guide sperm migration. Indeed, nematode oocytes attract sperm by releasing a mixture of different hormone-like chemicals called ‘prostaglandins’ (Hoang et al., 2013). Thus, the COMP-1 protein might control the ability of sperm to sense compounds like these, or to respond to and move towards them. If so, studying COMP-1 should dramatically deepen our understanding of how the control of sperm movement influences sperm competition (Pizzari and Parker, 2009).

Whether COMP-1 is a general component of the machinery for sperm guidance or movement, or plays a direct role in making some sperm more competitive than others, remains unknown. However, the ability to use nematode genetics to solve these problems has opened up a vast new area for research, and placed *C. elegans* at the center of this critical field. Moreover, further genetic screens might identify additional players that control sperm competition.
